# Black Talc-Based TiO_2_/ZnO Composite for Enhanced UV-Vis Photocatalysis Performance

**DOI:** 10.3390/ma14216474

**Published:** 2021-10-28

**Authors:** Huan Shuai, Jiao Wang, Xianguang Wang, Gaoxiang Du

**Affiliations:** 1 School of Materials Science and Technology, China University of Geosciences, Beijing 100083, China; shuaihuan@email.cugb.edu.cn; 2 School of Basic Education, Beijing Polytechnic College, Beijing 100042, China; 3 Jiangxi Mineral Resources Guarantee Service Center, Nanchang 330025, China

**Keywords:** black talc carrier, heterostructure, photocatalyst, TiO_2_, ZnO

## Abstract

Herein, using black talc as a carrier, a ternary black talc-TiO_2_/ZnO composite photocatalyst was prepared by the sol-gel method, and the effect of the black talc on the hetero-structure properties of the TiO_2_ and ZnO was systematically studied. The prepared composite photocatalyst showed an excellent degradation performance of the pollutant, where black talc plays an important role in promoting the interface interaction by enhancing the contact area between the TiO_2_ and ZnO. Moreover, the free carbon element doping in black talc favors the formation of more oxygen vacancies, thereby improving the response as a photocatalyst in visible light. In addition, the carbon in the black talc can also adsorb organic pollutants and enrich the surroundings of the photocatalyst with pollutants, so it further improves the catalytic efficiency of the photocatalyst. Under UV irradiation, the degradation rate of Rhodamine B on black talc-TiO_2_/ZnO was found 3.3 times higher than that of black talc-TiO_2_ with good stability.

## 1. Introduction

With the rapid development of the industries, organic pollutants in the air and water have been increased and brought a series of environmental problems [1,2,3] along with serious concern for human health [4]. Therefore, it is of great significance to investigate environmentally friendly, low-cost, facile-efficient pollutant treatment solutions including photocatalytic degradation, which offers cost-effective simple preparation and stable chemical properties devoid of secondary pollution [5,6,7,8]. As an important semiconductor material, TiO_2_ has broad application prospects in energy conversion, catalysts, etc. [9,10,11,12].

However, some issues with TiO_2_, such as being prone to agglomeration, low specific surface area, difficult to recycle, and having a high photo-generated electron–hole recombination rate and narrow range of wavelength for utilization of light, can limit its applications [13]. However, the construction of a heterojunction by coupling TiO_2_ with other semiconductor photocatalysts such as ZnO, CdS, etc. can be an effective way to accelerate the separation of electron–hole pairs [14]. Meanwhile, the anchoring of catalyst particles on a suitable substrate can increase the contact area between the photocatalyst and pollutants, thereby increasing the photocatalytic activity.

On the other hand, ZnO, a semiconductor material with a bandgap of 3.37 eV, can only absorb ultraviolet (UV) light with a wavelength less than 378 nm [15,16]. The photogenerated electrons in ZnO are easy to recombine with holes, which reduce the concentration of the carriers that can migrate towards the surface, resulting in relatively low quantum efficiency [17]. As per the literature, the photocatalytic activity can be improved by combining two different semiconductors such as TiO_2_ and ZnO to form heterojunction, which can expand the absorption spectrum from UV to visible light and improve the quantum efficiency by accelerating the transfer of the photogenerated electrons and holes between the interface [18,19].

**Scheme 1:**
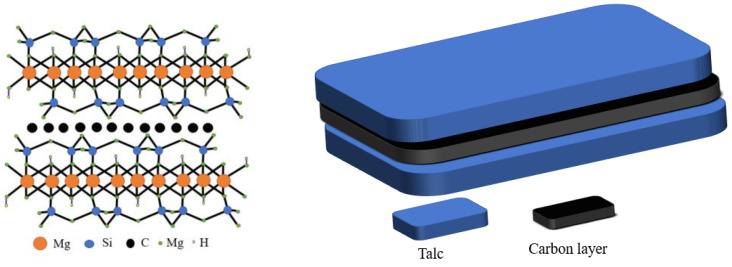
Schematic diagram of black talc structure.

In comparison with a single-phase photocatalyst, the composite photocatalyst possesses higher catalytic activity, but is difficult to recycle. A suitable carrier, such as black talc, can be used to immobilize the composite photocatalyst. Thus far, black talc is one of the few layered silicate minerals with a well-defined lamellar structure, which can facilitate the hosting of the photocatalysts. The structure is shown in Scheme 1. The abundant hydroxyl groups on the surface of the black talc favor the nucleation of the supported photocatalyst, improve their dispersion and stability, and increase their contact area to the surrounding matter. More importantly, black talc is a sandwich structure with a graphene-like carbon layer embedded between the talc layers [20,21], where organic pollutants can be preferentially adsorbed on the carbon layer naturally, thereby increasing the concentrations of the pollutants near composite photocatalyst and improving the efficiency of the catalytic. The carbon in black talc can further dope with ZnO to generate more oxygen vacancies, thereby improving its response against visible light. In addition, the carbon-doped ZnO can inhibit the recombination of the photo-generated carriers and increase quantum efficiency [22,23,24,25,26].

In order to increase specific surface area, catalytic efficiency, and adsorption of organic pollutants, and to achieve the synergistic catalytic effect, a new composite photocatalyst is an urgent requirement.

In this study, a black talc-TiO_2_/ZnO composite photocatalyst with smaller particle size and larger specific surface area is prepared by the sol-gel method to provide the combination of large pollutant absorption capacity and high catalytic efficiency. The formed heterojunction between TiO_2_ and ZnO provides significantly large charge transfer channels to favor high catalytic efficiency. The morphology, structure, and photocatalytic performance of the as-prepared black talc-TiO_2_/ZnO composites have been thoroughly characterized, and the effect of the black talc on the interaction between TiO_2_ and ZnO and output performance is systematically discussed. This study provides a new approach to synthesize novel and efficient hetero-structured photocatalysts in a facile and low-cost manner.

## 2. Materials and Methods

In this work, the materials and reagents were used as received, without further treatment. Black talc was purchased from Guangfeng, Jiangxi. Tetrabutyl titanate (TBOT), and zinc nitrate were purchased from Aladdin Reagent Co., Ltd. (Shanghai, China). The sulfuric acid, sodium hydroxide, and analytical pure ethanol were purchased from Beijing Chemical Plant (Beijing, China). Deionized water was used in the whole experimental process.

**Scheme 2:**
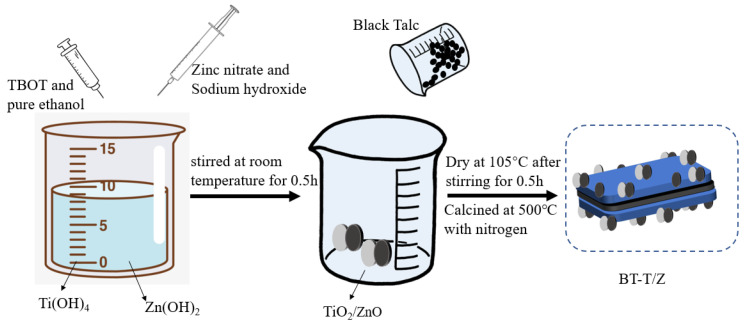
Synthesis strategy for BT-T/Z composite photocatalyst.

The black talc-TiO_2_/ZnO composite was prepared by the sol-gel method. In a typical synthesis process, a 10.0% sulfuric acid solution was added to 325 mesh black talc powder and stirred for 2 h. Following this, the mixture was filtered via a suction filter, and the product was washed with deionized water three times. Next, the product was dried at 105 °C for 24 h prior to use and labeled as BT. The BT sample was calcined at 500 °C in a furnace purged with oxygen flow to obtain calcined black talc (CBT). Then, 8.5 g of TBOT was dissolved in 30 mL of absolute ethanol and stirred thoroughly to obtain solution A. Thereafter, 10 g of BT sample was added to solution A under stirring conditions. Finally, the mixture was placed in an oven at 105 °C for completely dry powder, and it was calcined in a tube furnace purged with nitrogen flow at 500 °C for 2 h before. Thus, the black talc-supported titanium dioxide composite photocatalyst was obtained and labeled as BT-T. The black talc-TiO_2_/ZnO composite was prepared according to the following steps as shown in Scheme 2. Firstly, 5 g of zinc nitrate and 50 mL of sodium hydroxide solution (1 M) were evenly mixed and added to solution A, which was stirred for half an hour at room temperature. Afterward, the synthesized BT was added and stirred for half an hour before it was kept in an oven at 105 °C for drying. Finally, the dried product was placed in a tube furnace purged with nitrogen flow and calcined at 500 °C for 2 h, and the black talc supported titanium dioxide/zinc oxide composite photocatalyst was obtained and denoted as BT-T/Z.

## 3. Results

**Figure 1:**
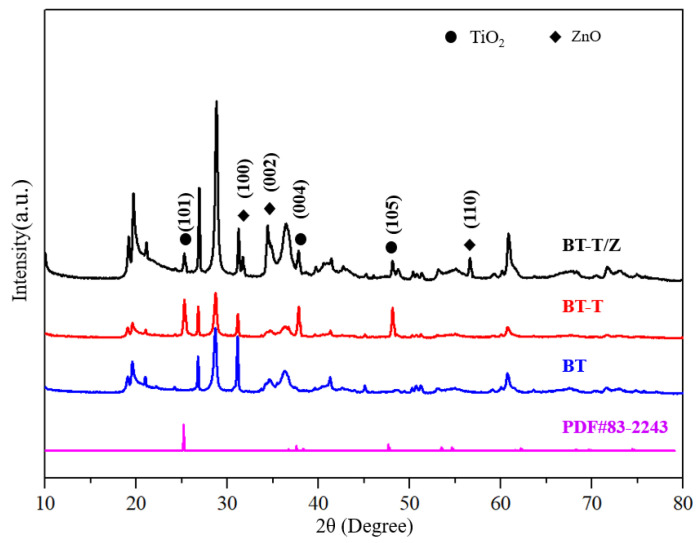
XRD diffraction pattern for photocatalytic material.

Figure 1 shows the XRD patterns for BT, BT-T, and BT-T/Z composite photocatalysts. The characteristic diffraction peaks for the anatase phase are observed in both BT-T and BT-T/Z samples at 25.3°, 37.8°, and 48.1°, which are attributed to the diffraction from (101), (104), and (200) planes of the anatase TiO_2_, respectively [27]. Importantly, no characteristic peak is observed for the rutile TiO_2_ phase. The results indicate that the anatase phase of the TiO_2_ has been successfully synthesized on black talc in both BT-T and BT-T/Z samples. The characteristic diffraction peaks for the ZnO are observed in the BT-T/Z sample at 31.7°, 34.4°, and 56.6° from the (100), (002), and (110) diffraction planes for ZnO, respectively. The results show that the ZnO phase has been successfully coated onto the composite material. Meanwhile, the relative content of the black talc, i.e., BT sample is reduced with the formation of the TiO_2_ and ZnO as the intensity of the characteristic peak becomes weak. However, all the diffraction peaks of the BT are stable, indicating that calcination at 500 °C cannot damage the black talc structure.

**Figure 2:**
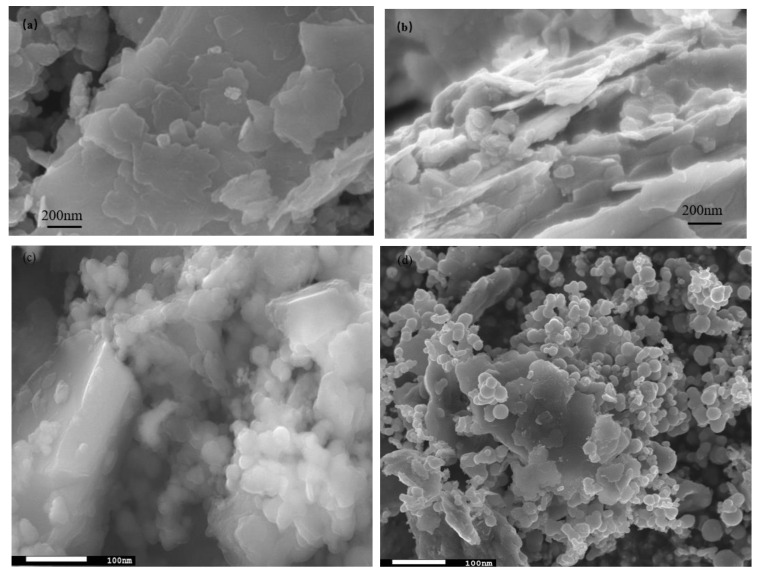
(**a**,**b**) SEM image of black talc, (**c**,**d**) SEM image of photocatalyst.

**Figure 3:**
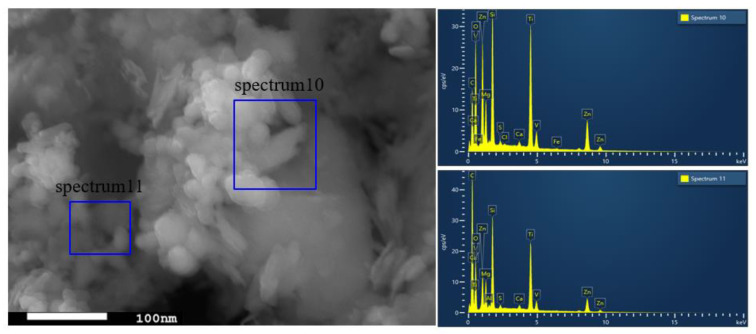
EDS element probing for photocatalyst.

**Figure 4:**
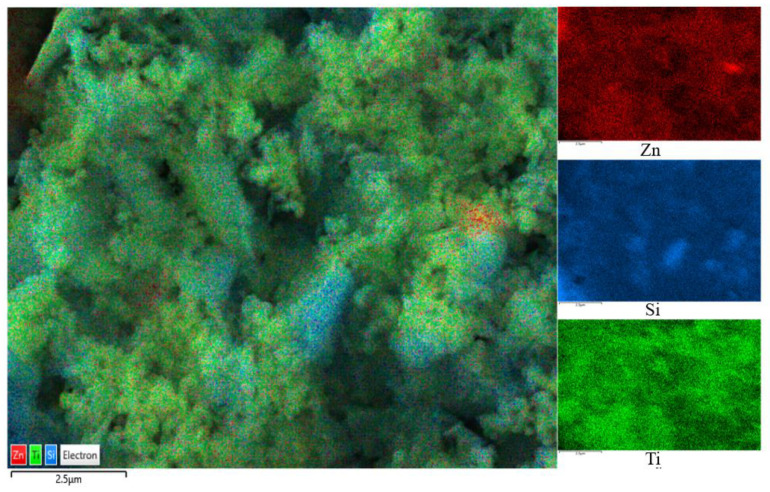
EDS element mapping for photocatalyst.

The SEM images are shown in Figure 2, where Figure 2a,b are captured for the BT sample while Figure 2c,d are captured for the photocatalyst BT-T/Z. It can be seen that the black talc exhibits a layered structure with small mineral particles of size less than 2 microns. The stacking of these flakes in the talc has a certain orientation along (001) planes in parallel to each other in a layered stack. From SEM images in Figure 2c,d, a large number of particles are agglomerated on the surface of the black talc mineral, especially at its edge, with a relatively uniform size of about 20 nm. However, the lamellar structure for the black talc can be clearly seen after agglomeration. The compositional analysis of the elements by EDS confirms the existence of both TiO_2_ and ZnO on the surface of the black talc mineral, depicting the composite phase. Figure 3 shows the EDS element probing for photocatalyst. And Figure 4 shows the EDS element mapping for photocatalyst.

**Figure 5:**
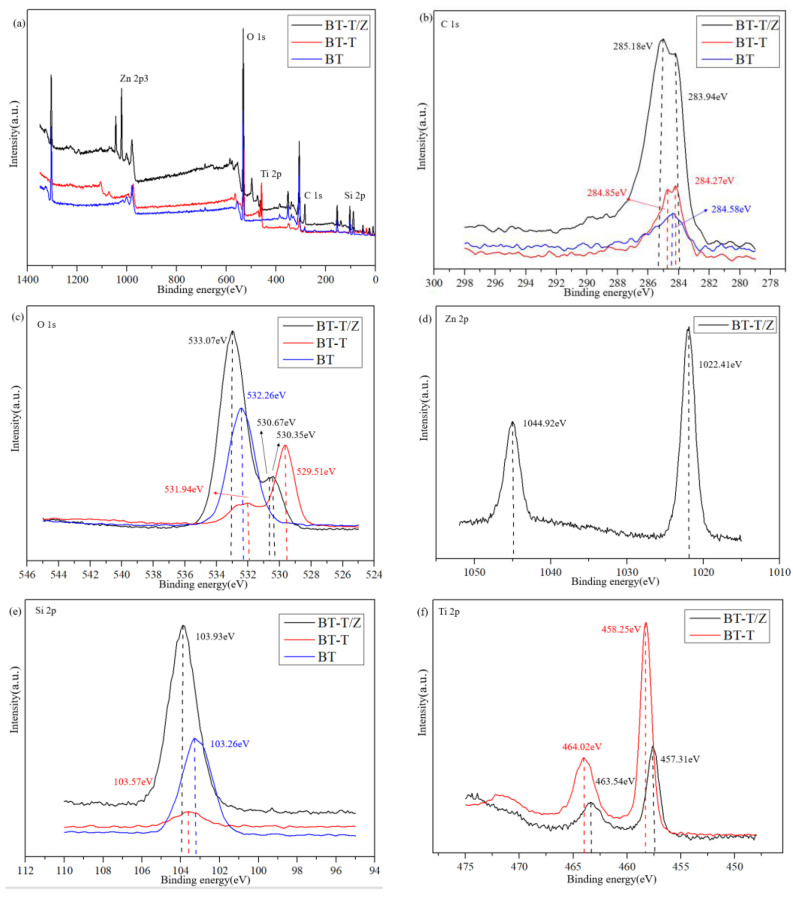
(**a**) XPS spectra of BT, BT-T, and BT-T/Z, (**b**) C 1s, (**c**) O 1s, (**d**) Zn 2p, (**e**) Si 2p, and (**f**) Ti 2p high-resolution energy spectrum.

The profile obtained from X-ray photoelectron spectroscopy (XPS) for the BT-T/Z sample shows five chemical elements, namely Zn, O, Ti, C, and Si, as shown in Figure 5a. Figure 5b shows C 1s peaks for the BT-T/Z. The characteristic peak at 285.18 eV confirms the existence of C–C bonds in the sample. The narrow C 1s peaks in the BT-T and BT also appear at nearly 284 eV. As compared with the BT sample, the binding energy for the C element in the BT-T/Z and BT-T increases slightly due to the bound state of carbon atoms in TiO_2_/ZnO heterojunction. In addition, the characteristic peak at 283.94 eV indicates that the formation of Zn–C bond on the black talc, i.e., C-doped ZnO [28,29].

Figure 5c shows the O 1s profile at 532.26 eV for Si-O-Si. The characteristic peaks at 529.51 eV and 530.35 eV are observed for Ti-O-Si, and that peak at 530.67 eV is attributed to the presence of oxygen defects or hydroxyl groups on the surface of the ZnO [30,31].

Figure 5d shows that the Zn 2p at 1022.41 eV is attributed to the Zn–O bond, while the Zn 2p1/2 peak appears at 1044.92 eV. The distance between the two peaks is observed at nearly 22 eV, corresponding to the featured profile of Zn^2+^ [32].

Figure 5e indicates that the Si 2p peak shifts to the blue end when the SiO_2_ in black talc hybridized with TiO_2_ and ZnO, confirming the effective combination of SiO_2_ with TiO_2_ and ZnO. Thus, a chemical bond is formed between TiO_2_/ZnO and black talc.

Figure 5f shows the peaking at 464.02 eV and 458.25 eV are corresponding to the characteristic peaks of the Ti 2p1/2 and Ti 2p3/2, respectively. A span of 5.8 eV is observed between the two peaks, indicating Ti in its oxidation state of Ti^4+^ [33,34]. Comparably, the characteristic peak of Ti 2p in the BT-T/Z is much weaker than that of the BT-T due to the coating of the ZnO on the surface of the BT-T/Z composite. As the electronegativity of Si atoms is higher than that of Ti atoms, it can induce positively charged holes to move towards the Si-O-Ti interface and promote the directional diffusion of the photogenerated electrons and holes. With the increase in the contact area between TiO_2_ and ZnO, a large number of electron transfer channels are generated at the interfaces due to the formation of TiO_2_/ZnO heterostructure.

**Figure 6:**
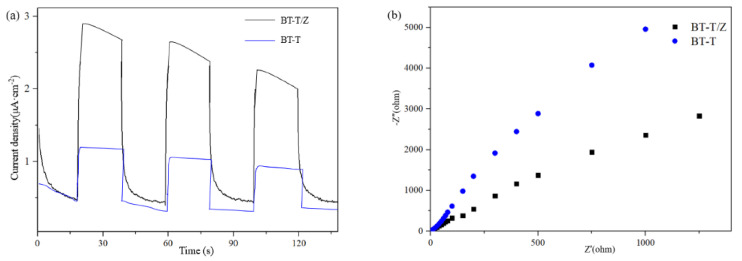
(**a**) Photoelectric conversion performances and (**b**) electrochemical impedance spectra.

In order to study the performance of the hetero-structure between TiO_2_ and ZnO, the photocurrent transient response and electrochemical impedance spectroscopy (EIS) were measured, and the results are shown as Figure 6a,b, respectively. It can reflect the charge separation efficiency and charge transfer resistance of the BT-T and BT-T/Z.

Figure 6a shows that the BT-T/Z sample exhibits a higher photocurrent transient intensity than that of the BT-T sample, indicating that the coexistence of TiO_2_ and ZnO is beneficial for the superior performance of the composite. Meanwhile, the TiO_2_/ZnO composite exhibits a smaller arc radius (Figure 6b) in the counterpart of the BT-T sample, showing smaller charge transfer resistance. This indicates that defects contribute to the increase in donor density and the decrease in charge transfer resistance. Therefore, the close contact between TiO_2_ and ZnO leads to the high mobility of the photogenerated electron-hole pair. The results confirm that the strong light response and small charge transfer resistance due to the formation of hetero-structure between the TiO_2_ and ZnO can lead to a better photocatalytic performance from BT-T/Z composite than BT-T sample.

Herein, Rhodamine B was used to mimic organic pollutants in the adsorption-degradation experiments. The determination of the degradation rate can be expressed as

η = (A_0_ − A)/A_0_ × 100% (1)

where η is the decolorization rate for Rhodamine B. The absorbance, A_0_, is determined for the rhodamine B solution before degradation, and A is the absorbance of the rhodamine B solution after degradation.

In a typical adsorption experiment, the four quartz test tubes were filled with 50 mL of rhodamine B solution (30 mg/L), respectively, and 50 mg of the BT, BT-T, and BT-T/Z are added to the mentioned three solutions, respectively, while the fourth was considered as a reference solution. Afterward, all the solutions were stirred at a constant speed with a magnet under dark conditions to ensure even mixing.

**Figure 7:**
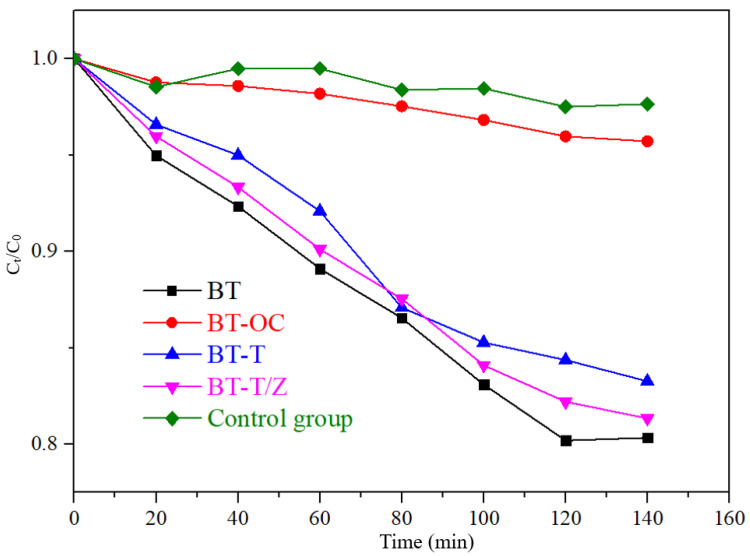
The adsorption effect of RhB on different samples under dark conditions.

The mixtures were sampled every 20 min, and the supernatants obtained by centrifuging were measured three times for each sample by UV-vis at 554 nm to determine the change in the concentration of rhodamine B in the supernatant. According to the formula (C_0_ − C)/C_0_, the degradation rate of the Rhodamine B in a solution can be evaluated, and the outcomes are shown in Figure 7.

As shown in Figure 7, under dark conditions, the compound Rhodamine B itself is relatively stable with only 3% decay in the concentration. The adsorption rate of CBT is 4.3%, which is slightly higher than that of the control group. The adsorption rate of 16.8% of the BT-T to rhodamine B is observed, and it is 18.7% for BT-T/Z, which is slightly lower than that of BT (19.7%) as the adhesion of TiO_2_ and ZnO affects the adsorption of Rhodamine B. After 120 min, the adsorption rate for each sample remains unchanged due to the saturation of the adsorption in the absence of photocatalytic degradation.

**Figure 8:**
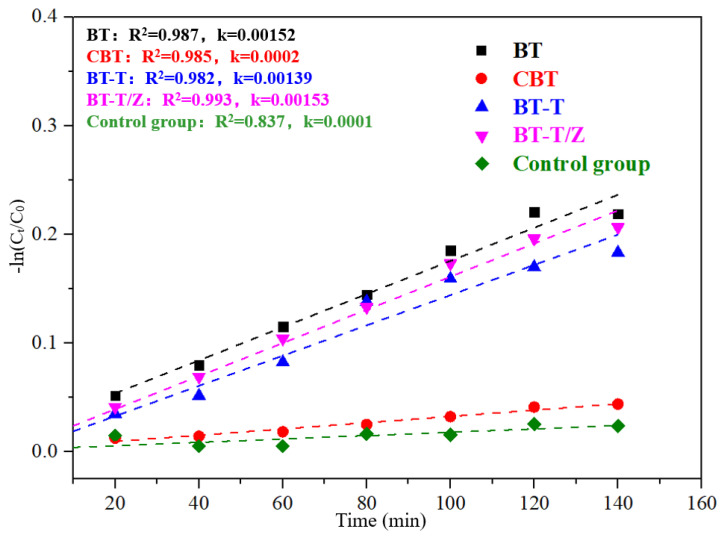
Kinetics fitting curves for the adsorption of Rhodamine B under dark conditions.

The physical adsorption of Rhodamine B solution to black talc follows the quasi-first-order kinetic equation, as shown in Figure 8, where the adsorption performance for each sample can be quantitatively evaluated through the apparent rate constant k.

−ln(C/C_0_) = kt (2)

The photocatalytic degradation experiment was carried out using a high-pressure mercury lamp with a power of 300 W irradiating on the BT-T and BT-T/Z samples with a dominant wavelength of 365 nm. The whole reactor was immersed into a water circulation system to keep the temperature at 18 °C during the reaction. The successive sampling was taken at an interval of 20 min, and each sample was tested three times to obtain an average value. The initial concentration (absorbance) for Rhodamine B is denoted as C_0_, and the concentration for the supernatant obtained at a different time is recorded as C so that the formula (C_0_ − C)/C_0_ can denote the degradation rate under this condition.

**Figure 9:**
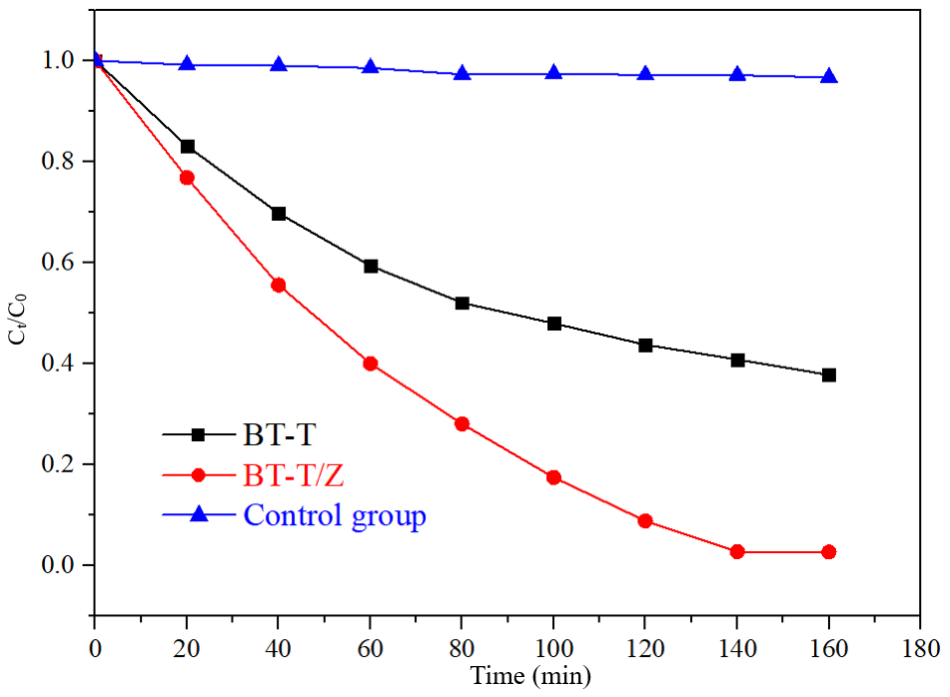
Degradation curves of Rhodamine B with the different photocatalyst under UV irradiation.

Figure 9 shows the degradation curves of Rhodamine B with the different photocatalyst under UV irradiation. Under UV irradiation, the concentration of Rhodamine B decreases less than 4% in the control group, indicating that Rhodamine B is quite stable under UV irradiation. The degradation rate reached 97.3% for BT-T/Z while 60% for BT-T after 140 min of irradiation. Obviously, the composite BT-T/Z showed the best photocatalytic performance, where TiO_2_ and ZnO possess a synergistic effect in the photodegradation of Rhodamine B.

**Figure 10:**
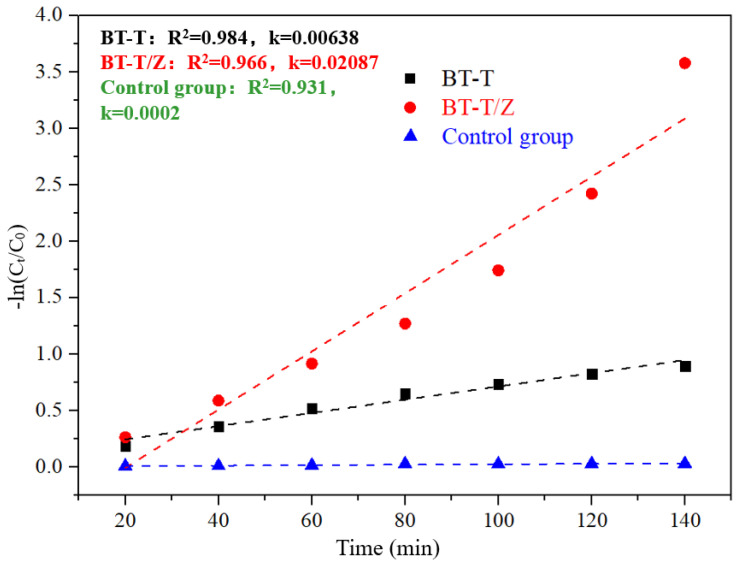
Kinetics fitting plots for the degradation of Rhodamine B under the irradiation with a wavelength of 365 nm.

Figure 10 shows thekinetics fitting plots for the degradation of Rhodamine B under the irradiation with a wavelength of 365 nm.Through fitting kinetics, the apparent degradation rate constant of 0.00638 is evaluated for BT-T while 0.02087 for the BT-T/Z, where the degradation efficiency of the BT-T/Z is 3.3 times higher than that of the BT-T sample. This result confirms again that BT-T/Z is an excellent photocatalyst for organic pollutant degradation.

**Figure 11:**
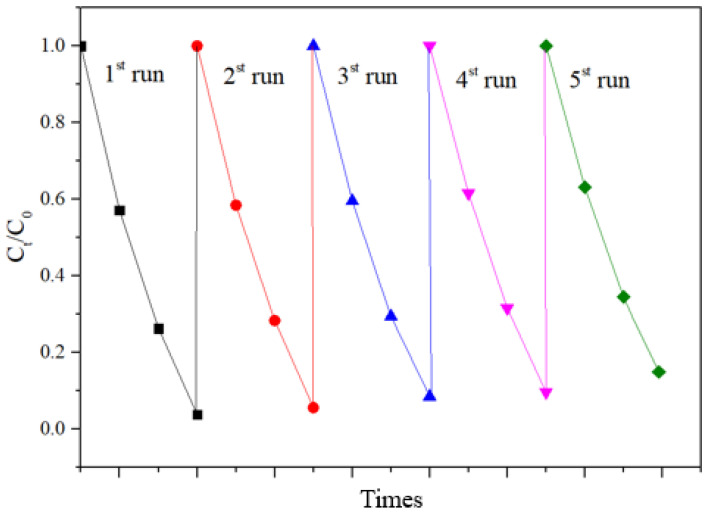
Cycling degradation test of Rhodamine B on BT-T/Z.

In order to further test the stability of the prepared photocatalyst, cyclic degradation tests were conducted. The experimental results are shown in Figure 11. The experimental results show that the adsorption–degradation rate for Rhodamine B being catalyzed by BT-T/Z was still around 88% after five cycles, indicating good stability and reusability.

## 4. Discussion

**Scheme 3:**
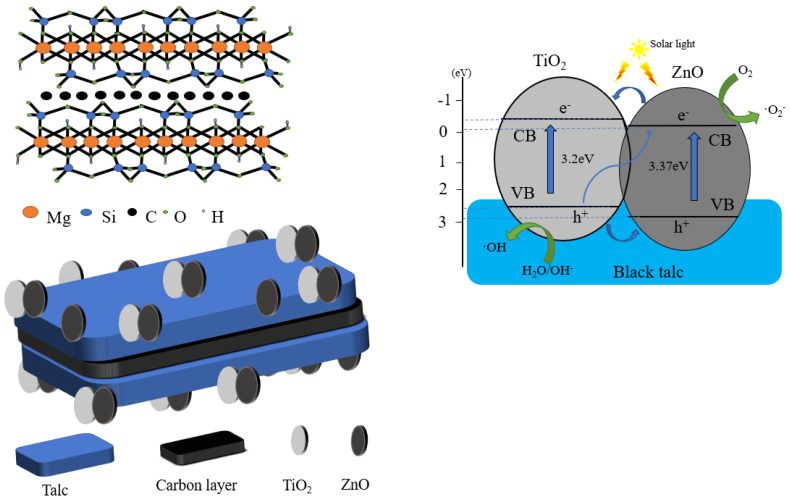
The improved photocatalysis mechanism for BT-T/Z composite under photo-irradiation.

As shown in Scheme 3, an efficient hetero-structure is formed between the TiO_2_ and ZnO through their close interfacial combination with the substrate of BT. Under the photo-irradiation, the electrons in the valence band of the TiO_2_ and ZnO are excited and transfer to their conduction band, whereas the holes remain in the valence band. The excited electrons in the ZnO recombine with the holes in the TiO_2_ and weaken the electron–hole recombination extent for the TiO2, resulting in the majority of electrons surviving in the conduction band of the TiO2. Meanwhile, the holes accumulated in the valence band of the TiO2 take part in the oxidation reaction. The formation of hetero-structure between the TiO2 and ZnO can significantly improve the separation and transport efficiency of the photogenerated carriers, which is one of the important factors, causing an improved photocatalytic redox performance for BT-T/Z in the counterparts of the BT-T. The black talc has abundant surface hydroxyl groups and high surface activity, playing a synergic role in improving photocatalytic performance. Compared with other studies [35,36,37], this experiment cleverly combined the construction of heterojunction and the loading of mineral materials to prepare a photocatalyst with good performance.

## 5. Conclusions

In summary, firstly, the presence of black talc reduces the particle size of the photocatalyst and increases its specific surface area. Therefore, more TiO_2_ are exposed to contact and combine with ZnO, which causes more photocarriers to transfer pathways at their interface and favors an efficient photocarrier migration crossing at the interface between the TiO_2_ and ZnO.

Moreover, a graphene-like carbon layer in black talc can adsorb and enrich organic pollutants, thereby increasing the catalytic efficiency. Finally, the free carbon in the black talc may participate in the doping, and it favors the formation of more oxygen vacancies and improves its response in visible light.

Finally, BT-T/Z composite photocatalyst has been prepared through the sol-gel method. Under UV irradiation, the degradation rate of Rhodamine B catalyzed by BT-T/Z composite was found 3.3 times higher than that of BT-T. The improved photocatalytic performance for BT-T/Z can be attributed to the synergistic effect of the characteristic adsorption of black talc and the formation of a high-efficiency TiO_2_/ZnO hetero-structure. The existence of black talc can increase the specific surface area and light absorption and therefore further improve its photocatalytic efficiency. More importantly, the black talc can adsorb and enrich the pollutants around the photocatalyst, thereby increasing its catalytic efficiency. Consequently, black talc can be used as an effective carrier with multiple functions to improve the performance of the semiconductor photocatalysts. We believe that black talc can also be introduced into other semiconductor systems to form new high-efficiency composite photocatalysts, facilitating the development of high-efficiency and low-cost photocatalysts.

## Data Availability

The data presented in this study are available on request from the corresponding author.
